# Fractional Flow Reserve to Assess Coronary Artery Disease in Patients with Severe Aortic Stenosis Undergoing Transcatheter Aortic Valve Implantation: Long-Term Outcomes

**DOI:** 10.1016/j.shj.2023.100179

**Published:** 2023-04-18

**Authors:** Juva Benseba, Julien Mercier, Thomas Couture, Laurent Faroux, Laurence Bernatchez, Mélanie Côté, Vassili Panagides, Jules Mesnier, Siamak Mohammadi, Éric Dumont, Dimitri Kalavrouziotis, Sandra Hadjadj, Jonathan Beaudoin, Robert DeLarochellière, Josep Rodés-Cabau, Jean-Michel Paradis

**Affiliations:** a Department of Cardiology, Quebec Heart and Lung Institute, Quebec, Canada; b Department of Cardiac Surgery, Quebec Heart and Lung Institute, Quebec, Canada

**Keywords:** Aortic valve stenosis, Coronary artery disease, Fractional flow reserve, Intracoronary adenosine, Transcatheter aortic valve implantation

## Abstract

**Background:**

The long-term outcomes of patients undergoing functional assessment of coronary lesions with fractional flow reserve (FFR) while awaiting transcatheter aortic valve implantation (TAVI) are unknown. Data on the safety of intracoronary adenosine use in this setting are scarce. The objectives of this study were to describe (1) the long-term outcomes based on the coronary artery disease (CAD) assessment strategy used and (2) the safety of intracoronary adenosine in patients with severe aortic stenosis (AS).

**Methods:**

1023 patients with severe AS awaiting TAVI were included. Patients were classified according to their CAD assessment strategy: angiography guided or FFR guided. Patients were further subdivided according to the decision to proceed with percutaneous coronary intervention (PCI): angiography-guided PCI (375/1023), angiography-guided no-PCI (549/1023), FFR-guided PCI (50/1023), and FFR-guided no-PCI (49/1023). Patients were followed up for the occurrence of major adverse cardiac and cerebrovascular events (MACCEs).

**Results:**

At a mean follow-up of 33.7 months, we observed no significant differences in terms of major adverse cardiovascular and cerebrovascular events (MACCE) in the angiography-guided group (42.4%) compared with the FFR-guided group (37.4%) (*p* = 0.333). When comparing outcomes of the FFR-guided no-PCI group (32.7%) with the angiography-guided PCI group (46.4%), no significant difference was noted (*p* = 0.999). Following intracoronary adenosine, a single adverse event occurred.

**Conclusions:**

In this population, intracoronary adenosine is safe and well tolerated. We found no significant benefit to an FFR-guided strategy compared with an angiography-guided strategy with respect to MACCEs. Although clinically compelling, avoiding the procedural risks of PCI by deferring the intervention in functionally insignificant lesions failed to show a statistically significant benefit.

## Introduction

Over the past several years, transcatheter aortic valve implantation (TAVI) arose as an alternate, noninferior, and possibly favorable therapeutic option for selected patients with severe aortic stenosis (AS) with extreme,^1^^,^^2^ high,^3^ intermediate,^4^ and even low surgical risk.^5^ The prevalence of coronary artery disease (CAD) in this population ranges from ∼25%^5^ to ∼80%.^1^ This disparity can be explained by different definitions and methods to assess CAD.^6^ At present, there is no universally accepted approach for the assessment and treatment of CAD in patients with severe AS before TAVI. As suggested in the 2020 American Heart Association/American College of Cardiology guidelines,^7^, ^8^, ^9^ percutaneous coronary intervention (PCI) is recommended in patients undergoing valve replacement if significant CAD (>70% diameter stenosis in major coronary arteries or >50% diameter stenosis in left main) is present.

Fractional flow reserve (FFR) is the established standard of care for the functional assessment of lesion severity in intermediate-grade stenosis (including left main) and multivessel CAD in patients without valvular disease.^10^, ^11^, ^12^, ^13^ The accepted cutoff of ≤0.80 suggests hemodynamic relevance. The main clinical trials^11^^,^^12^ studying the use of FFR excluded patients with severe AS because of the presumed incapacity to produce maximal hyperemia. Combined with concerns about the safety of adenosine in patients with severe AS, FFR initially presented itself as an undependable strategy.^6^ Since then, the use of FFR in severe AS is described in current 2020 American Heart Association/American College of Cardiology guidelines for the management of valvular heart disease,^9^^,^^14^ but the efficacy, reliability, and superiority of FFR measurement in the specific population of severe AS patients awaiting TAVI are yet to be validated.^15^^,^^16^

Data from observational studies suggest validity of FFR guidance as well as favorable outcomes in small cohorts of patients with severe AS awaiting TAVI at short-term follow-up.^17^^,^^18^

Furthermore, the safety of intravenous adenosine has only been studied in a small number of patients with severe AS,^14^ and its comparability with intracoronary adenosine is proven^19^, ^20^, ^21^, ^22^ in patients without AS. Data on the safety of intracoronary adenosine in patients with severe AS are very scarce.

Our study aims to investigate the long-term outcomes of patients with severe AS according to their CAD assessment strategy before TAVI; FFR-guided vs. angiography-guided. We further subdivided and explored the differences between 4 subgroups of patients considering the decision to proceed with PCI following the strategy of CAD assessment used (angiography-guided PCI, angiography-guided no-PCI, FFR-guided PCI, FFR-guided no-PCI). Another objective was to establish the safety of intracoronary adenosine use in patients with severe AS.

## Methods

**Figure 1:**
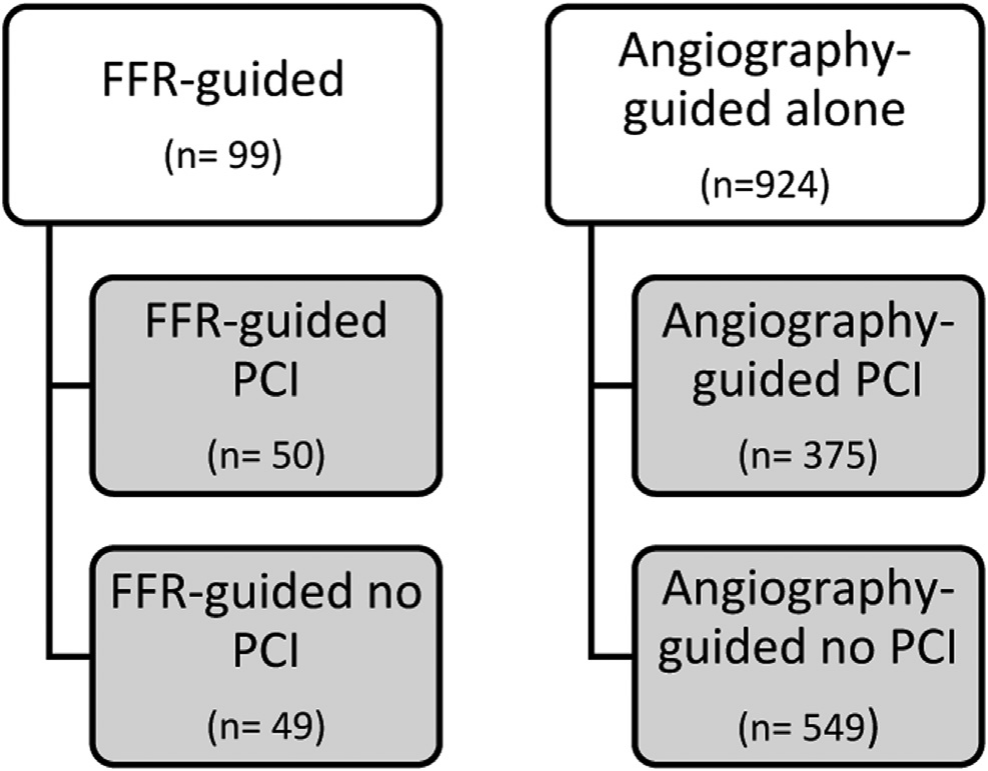
**Groupcharacteristics**. Abbreviations: FFR, fractional flow reserve; PCI, percutaneous coronary intervention.

All patients included in this study had severe symptomatic AS and a clinical indication to proceed with TAVI after the evaluation by the dedicated heart team. Patients with at least 1 FFR measurement of an intermediate (between 50% and 70%) coronary lesion identified during the pre-TAVI coronary angiogram were classified in the FFR-guided group. Patients who underwent angiography without any FFR assessment were classified in the angiography-guided group. We further subdivided these 2 groups according to the decision to proceed with PCI (Figure 1). All patients were treated with TAVI between November 2012 and November 2019, and follow-up data for the primary and secondary outcomes were collected up until July 2021.

The primary outcome was the incidence of major adverse cardiac and cerebrovascular events (MACCEs) defined as the composite of all-cause mortality, stroke/transient ischemic attack, myocardial infarction (MI), and elective PCI. Secondary outcomes comprised each component of the primary outcome composite separately, cardiac death, and in-hospital adverse events after TAVI (death, stroke/transient ischemic attack, MI, major bleeding, major vascular complication, acute kidney injury [AKI], and pacemaker implantation). All endpoints were categorized using the Valve Academic Research Consortium-2 (VARC-2) criteria.^23^

Finally, after an individual chart review, data on all adverse events during intracoronary adenosine infusion were collected.

This retrospective observational cohort study was approved by the ethics committee of the Quebec Heart and Lung Institute. All patients eligible for the protocol had provided their written informed consent for all the procedures involved (coronary angiogram, FFR, PCI, and TAVI).

### Study Groups

A total of 1023 patients were included in this retrospective study and classified according to the strategy of CAD assessment (FFR or angiography alone). Baseline characteristics including major cardiovascular risk factors and history of CAD were collected alongside baseline surgical risk scores (short-term risk and log-EuroScore). Echocardiographic features including left ventricular ejection fraction, aortic valve gradients, and pre-TAVI valve area, as well as severity of aortic regurgitation after TAVI, were also gathered (see Table 1).

**Table 1:** Baseline characteristics

Variables	Angio-guided PCI (n = 375)	Angio-guided no-PCI (n = 549)	FFR-guided PCI (n = 50)	FFR-guided no-PCI (n = 49)	*p* Value
Baseline characteristics					
Age	81 (76-85)	80 (73-85)†	82 (75-86)	82 (75-87)	0.036
Male sex	245 (65.3)	280 (51.1)†	24 (48.0)	27 (55.1)	<0.01
BMI	27 (24-30)	27 (24-31)	28 (23-31)	25 (24-29)	0.623
COPD	89 (23.7)	155 (28.2)	14 (28.0)	12 (24.5)	0.475
Creatinine (mmol/l)	100.5 (80.0-129.0)	92.0 (76.0-118.0)†	114.0 (85.0-140.0)	112.0 (83.0-135.0)	<0.01
eGFR <60	191 (50.9)	247 (45.0)	22 (44.0)	26 (53.1)	0.260
Dialysis	9 (2.4)	9 (1.6)	1 (2.0)	0 (0)	0.643
NYHA III-IV	226 (60.3)	337 (61.4)	31 (62.0)	24 (49.0)	0.383
Diabetes	156 (41.6)	182 (33.2)	16 (32.0)	16 (32.7)	0.058
Hypertension	332 (88.5)	458 (83.4)	43 (86.0)	40 (81.6)	0.157
Previous MI	89 (23.7)	48 (8.7)†	5 (10.0)†	8 (16.3)	<0.01
Previous PCI	363 (96.8)	0 (0)†	49 (98.0)‡	17 (34.7)†^,^‡^,^§	<0.01
Previous CABG	147 (39.2)	146 (26.6)†	4 (8.0)†^,^‡	6 (12.2)†	<0.01
PVD	111 (29.6)	129 (23.5)	9 (18.0)	12 (24.5)	0.113
Previous pacemaker	83 (22.1)	90 (16.4)	2 (4.0)†	6 (12.2)	<0.01
Atrial fibrillation	132 (35.2)	182 (33.3)	12 (24.0)	11 (22.4)	0.157
Previous stroke/TIA	59 (15.7)	56 (10.2)	6 (12.0)	7 (14.3)	0.095
Log_EUROSCORE	17.3 (9.6-27.0)	13.7 (8.1-24.1)†	12.6 (7.9-19.7)†	15.7 (10.7-26.9)	<0.01
STS score	5.1 (3.6-7.6)	4.4 (3.0-7.0)	5.4 (3.3-7.4)	4.6 (3.3-6.8)	0.409
Echocardiographic features pre-TAVI					
LVEF (%)	52 ± 12	54 ± 12	54 ± 13	54 ± 15	0.200
Aortic valve max gradient (mmHg)	64 (49-80)	70 (53-90)†	76 (59-92)†	64 (59-85)	<0.01
Aortic valve mean gradient (mmHg)	37 (29-47)	41 (31-55)†	45 (35-60)†	39 (34-55)	<0.01
AVA (cm^2^)	0.70 (0.60-0.80)	0.68 (0.54-0.83)	0.67 (0.52-0.77)	0.65 (0.50-0.78)	0.051
Procedural characteristics					
Approach					
Transfemoral	225 (60.0)	367 (66.8)	35 (70.0)	30 (61.2)	0.140
Nontransfemoral	150 (40.0)	182 (33.2)	15 (30.0)	19 (38.8)	
Transapical	60 (16.0)	75 (13.7)	5 (10.0)	9 (18.4)	
Transcarotid	69 (18.4)	76 (13.8)	7 (14.0)	6 (12.2)	
Transaortic	19 (5.1)	30 (5.5)	3 (6.0)	4 (8.2)	
Trans-subclavian	2 (0.5)	1 (0.2)	0 (0)	0 (0)	
Echocardiographic features post-TAVI					
LVEF (%)	52 ± 12	54 ± 12	53 ± 12	53 ± 14	0.052
Aortic valve max gradient (mmHg)	18.4 (13.6-23.0)	20.0 (15.0-28.0)†	18.0 (16.0-26.0)	17.0 (14.0-22.8)	<0.01
Aortic valve mean gradient (mmHg)	10.0 (7.0-12.0)	11.0 (8.0-14.5)†	10.0 (8.0-13.0)	9.3 (7.2-13.0)	<0.01
AVA (cm^2^)	1.50 (1.30-1.90)	1.50 (1.20-1.90)	1.50 (1.30-1.90)	1.50 (1.20-1.90)	0.704
Aortic regurgitation					
Moderate/severe (2-3/4)	96 (25.6)	145 (26.4)	11 (22.0)	14 (28.6)	0.480
Severe (4/4)	0 (0)	2 (0.4)	0 (0)	0 (0)	0.338

Continuous variables are reported as median (interquartile range); categorical variables are reported as n, (%).

AVA, aortic valve area; BMI, body mass index; CABG, coronary artery bypass graft; COPD, chronic obstructive pulmonary disease; eGFR, estimated glomerular filtration rate; LVEF, left ventricular ejection fraction; MI, myocardial infarction; NYHA, New York Heart Association; PCI, percutaneous coronary intervention; PVD, peripheral vascular disease; STS, short-term risk; TAVI, transcatheter aortic valve implantation; TIA, transient ischemic attack.

† Different from the angio-guided PCI group, *p* < 0.05.

‡ Different from the angio-guided no-PCI group, *p* < 0.05.

§ Different from the FFR-guided PCI group, *p* < 0.05.

All TAVI procedures were performed under our standard institutional clinical protocols. The choice of the transcatheter heart valve was left to the operator’s discretion. Balloon-expandable valves (SAPIEN XT, SAPIEN 3, and SAPIEN ULTRA) (Edwards Lifesciences, Irvine, California), as well as self-expandable bioprostheses (Corevalve, Evolut R, Evolut PRO) (Medtronic, Minneapolis, Minnesota) were implanted in this study.

### CAD Assessment

#### Coronary Angiography

Patients included in our study all had a level 1 class of recommendation to perform coronary angiography in the setting of their pre-TAVI workup.^9^ Coronary angiography was performed using the standard percutaneous radial or femoral approach. Severity of the CAD was assessed qualitatively by experienced interventional cardiologists. All coronary angiographies were then discussed during our weekly heart team meetings to decide on the best CAD assessment and management strategies. Given the longitudinal nature of this retrospective study, the decision to proceed with PCI or FFR measurement was based on the “at the time” standard practice. Angiographic characteristics of coronary lesions according to the treatment group are presented in Table 2.

**Table 2:** Pre-TAVI coronary angiography characteristics

Variables	Angio-guided PCI (n = 375)	FFR-guided PCI (n = 50)	FFR-guided no-PCI (n = 49)	*p* Value
No. of total lesions, n (%)	1378	218	135	-
No. of lesions >50%	1013	140	68	-
CAD severity				
Single-VD n (%)	126 (33.6)	24 (48.0)∗	27 (55.1)∗	<0.01
2-VD, n (%)	76 (20.3)	20 (40.0)∗	10 (20.4)†	<0.01
3-VD, n (%)	14 (3.7)	6 (12.0)∗	12 (24.5)∗	<0.01
Vessel disease				
LM, n (%)	24 (6.4)	4 (8.0)	8 (16.3)	0.061
LAD, n (%)	114 (30.4)	38 (76.0)∗	30 (61.2)∗	<0.01
LCx, n (%)	84 (22.4)	19 (38.0)∗	18 (36.7)∗	<0.01
RCA, n (%)	97 (25.9)	22 (44.0)∗	23 (46.9)∗	<0.01
Vein graft, n (%)	44 (11.7)	1 (2.0)	4 (8.2)	0.09
LIMA, n (%)	47 (12.5)	0 (0)∗	0 (0)∗	<0.01
No. of lesions treated, n (%)	391	90	-	-
No. of stents implanted, n (%)	335	69		-
QCA pre-PCI				
Mean DS, %	45 ± 21	59 ± 10∗	51 ± 7†	<0.01

CAD, coronary artery disease; DS, diameter stenosis; FFR, fractional flow reserve; LAD, left anterior descending; LCx, left circumflex; LIMA, left internal mammary artery; LM, left main; PCI, percutaneous coronary intervention; QCA, qualitative coronary angiography; RCA, right coronary artery; TAVI, transcatheter aortic valve implantation; VD, vessel disease.

∗ Different from the angio-guided PCI group, *p* < 0.05.

† Different from the FFR-guided PCI group, *p* < 0.05.

#### FFR Measurements

In selected patients with at least 1 intermediate coronary lesion, FFR measurements were realized. After normalization in the aorta, a pressure wire (PressureWire X [Abbott Vascular, Minneapolis, Minnesota] or Optowire [Opsens, Quebec, Canada]) was positioned distally to the intermediate coronary artery stenosis. Hyperemia was observed after administration of intracoronary adenosine as boluses (210-300 μg for the left coronary artery and between 90 and 210 μg for the right coronary artery). Given the presence of severe AS in all patients, nitroglycerin was not administered. After each recording, the pressure wire was brought back to the tip of the guiding catheter to ensure that no pressure drift had occurred. If a pressure wire drift was present, all measurements were repeated. The clinical decision to perform myocardial revascularization with PCI was made when the FFR was less than or equal to 0.80, a value selected based on the results of previous trials. Patients with an FFR of more than 0.80 in a vessel received the best available medical therapy for CAD. Of note, in our study, all FFR-positive lesions were addressed with PCI. Data on vessel disease, artery FFR, adenosine route of administration, and dose are available in Table 3.

**Table 3:** FFR-guided assessment characteristics

Variables	FFR-guided (n = 99)	FFR-guided PCI (n = 50)	FFR-guided no-PCI (n = 49)	*p* Value
Mean pd/pa value	0.88 ± 0.08	0.83 ± 0.07	0.93 ± 0.04	<0.001
Mean FFR value	0.82 ± 0.08	0.75 ± 0.05	0.88 ± 0.05	<0.001
No. of total lesions treated, n (%)	90	90	-	-
Vessel disease				
LM, n (%)	12 (12.1)	4 (8.0)	8 (16.3)	0.204
LAD, n (%)	68 (68.7)	38 (76.0)	30 (61.2)	0.113
LCx, n (%)	37 (37.4)	19 (38.0)	18 (36.7)	0.896
RCA, n (%)	45 (45.5)	22 (44.0)	23 (46.9)	0.769
Vein graft, n (%)	5 (5.1)	1 (2.0)	4 (8.2)	0.204
LIMA, n (%)	-	-	-	-
Artery FFR				
LM, n (%)	7 (7.1)	2 (4.0)	5 (10.2)	0.046
LAD, n (%)	51 (51.5)	33 (66.0)	18 (36.7)	
LCx, n (%)	19 (19.2)	6 (12.0)	13 (26.5)	
RCA, n (%)	21 (21.2)	9 (18.0)	12 (24.5)	
Vein graft, n (%)	1 (1.0)	0 (0)	1 (2.0)	
LIMA, n (%)				
Syntax score (mean)	11.7 ± 7.5	12.9 ± 7.2	10.4 ± 7.6	0.051
Residual syntax score (mean)	5.5 ± 6.9	2.3 ± 4.7	8.8 ± 7.3	<0.001
Adenosine use				
Route of administration				0.029
Intracoronary adenosine %	91 (91.9)	43 (86.0)	48 (98.0)	
Intravenous adenosine %	0 (0)	0 (0)	0 (0)	
No IC or IV adenosine %	8 (8.1)	7 (14.0)	1 (2.0)	0.378
Mean adenosine dose	270.3 ± 89.4	264.4 ± 112.2	275.5 ± 63.8	0.569
RCA (n = 22)	239.1 ± 114.1	234.4 ± 159.5	242.3 ± 75.9	0.893
LM (including Cx, LAD, Bx) (n = 71)	280.4 ± 78.0	272.4 ± 97.6	287.8 ± 54.5	0.419

FFR, fractional flow reserve; IC, intracoronary; IV, intravenous; LAD, left anterior descending; LCx, left circumflex; LIMA, left internal mammary artery; LM, left main; PCI, percutaneous coronary intervention; RCA, right coronary artery.

### Statistical Analysis

Categorical variables were reported as percentages, and continuous data as mean ± standard deviation or median (interquartile range), depending on their distribution. Baseline and procedural variables were compared using generalized mixed models. Generalized mixed models were used to compare clinical outcomes between groups while adjusting for intergroup differences in baseline characteristics. Clinical event rates were also summarized by using the Kaplan-Meier estimates, and comparisons between groups were performed with the log rank test. A Cox proportional-hazards regression model that included all baseline variables with a *p* value of 0.10 or less for the between-group comparison was used to perform an adjusted analysis of survival. The resulting curves were plotted for each subgroup for comparison. Adjusted survival curve analyses were conducted using the statistical package SAS version 9.4 (SAS Institute Inc, Cary, North Carolina).

## Results

### Angiography-Guided vs. FFR-Guided

Of the 1023 patients included in this retrospective study, 924 had a CAD assessment strategy based on angiography alone while 99 patients also had a functional assessment of at least 1 intermediate coronary lesion during the pre-TAVI angiogram.

**Figure 2:**
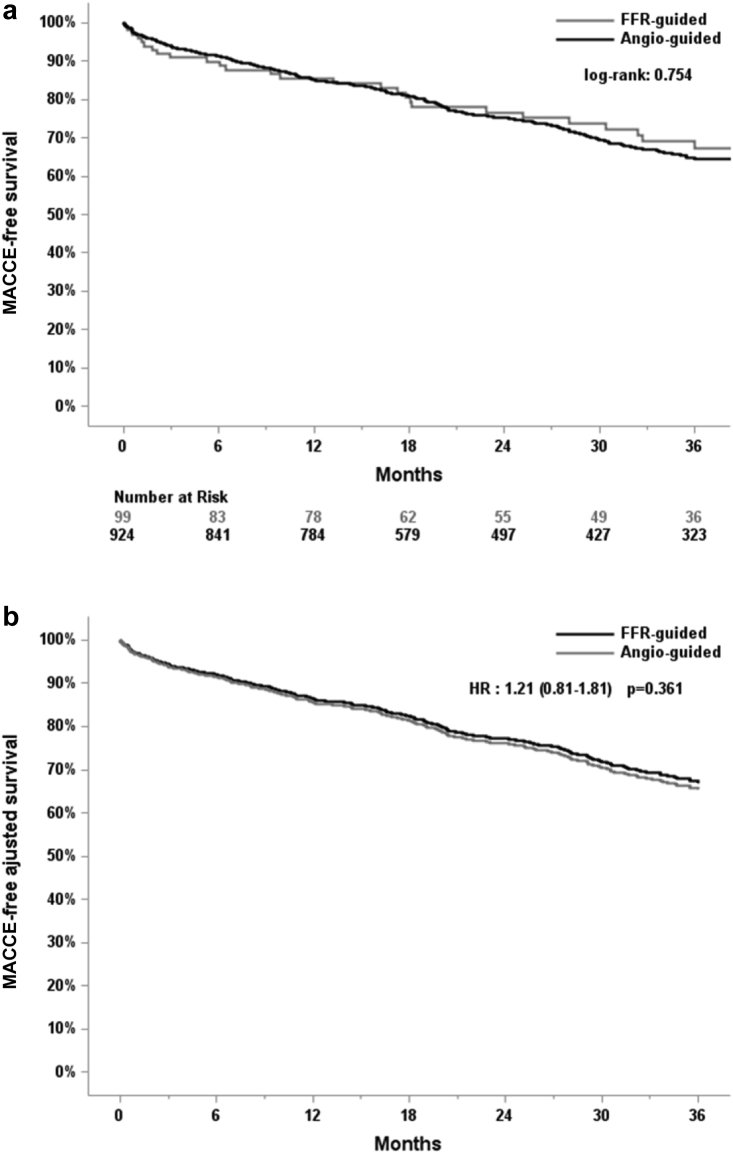
(a) MACCE-free survival and (b) MACCE-free adjusted survival for the primary outcome in the angiography-guided group vs. FFR-guided group. Abbreviations: FFR, fractional flow reserve; MACCE, major adverse cardiac and cerebrovascular events.

For in-hospital adverse events (death, stroke, MI, major bleeding, major vascular complication, AKI, new pacemaker), no significant differences were observed between the angiography-guided and the FFR-guided groups (Table 4). At a mean follow-up of 33.7 months, we observed no significant differences in terms of MACCE in the angiography-guided strategy group (392/924 patients; 42.4 %) in comparison to the FFR-guided strategy group (37/99 patients; 37.4%) (*p* = 0.333) (Table 5). MACCE-free survival Kaplan-Meier curves for the primary outcome are presented in Figure 2. For all secondary outcomes, no significant differences between the 2 groups were observed.

**Table 4:** In-hospital outcomes (post-TAVI)

In-hospital follow-up	Angio-guided PCI (n = 375)	Angio-guided no-PCI (n = 549)	FFR-guided PCI (n = 50)	FFR-guided no-PCI (n = 49)	Unadjusted *p* value	Adjusted *p* value†
MACCE, n (%)	29 (7.7)	36 (6.6)	5 (10.0)	5 (10.2)	0.643	0.928
Death, n (%)	13 (3.5)	14 (2.6)	2 (4.0)	4 (8.2)	0.221	0.577
Stroke/TIA	9 (2.4)	17 (3.1)	1 (2.0)	2 (4.1)	0.853	0.964
MI, n (%)	13 (3.5)	12 (2.2)	2 (4.0)	1 (2.0)	0.616	0.623
Major bleeding, n (%)	35 (9.3)	33 (6.0)	5 (10.0)	3 (6.1)	0.249	0.286
Major vascular complication, n (%)	25 (6.7)	21 (3.8)	3 (6.0)	1 (2.0)	0.197	0.155
Acute kidney injury, n (%)	46 (12.3)	47 (8.8)∗	4 (8.0)	4 (8.2)	0.247	0.007
New pacemaker	62 (16.5)	77 (14.0)	5 (10.0)	8 (16.3)	0.547	0.426
Procedural MI, n (%)	13 (3.5)	10 (1.8)	2 (4.0)	1 (2.0)	0.420	0.972

CABG, coronary artery bypass graft; FFR, fractional flow reserve; MACCE, major adverse cardiac and cerebrovascular events; MI, myocardial infarction; PCI, percutaneous coronary intervention; TAVI, transcatheter aortic valve implantation; TIA, transient ischemic attack.

∗ Different from the angio-guided PCI group, adjusted *p* < 0.05.

† Adjusted for age, gender, creatinine, previous MI, previous PCI, previous CABG, previous pacemaker.

**Table 5:** Outcomes (after TAVI) at long-term follow-up

33.7 months median follow-up	Angio-guided PCI (n = 375)	Angio-guided no-PCI (n = 549)	FFR-guided PCI (n = 50)	FFR-guided no-PCI (n = 49)	Unadjusted *p* value	Adjusted *p* value‡
MACCE, n (%)	174 (46.4)	218 (39.7)	21 (42.0)	16 (32.7)	0.118	0.239
Death, n (%)	144 (38.4)	185 (33.7)	16 (32.0)	13 (26.5)	0.259	0.221
Cardiac death, n (%)	44 (11.7)	60 (10.9)	5 (10.0)	5 (10.2)	0.966	0.997
Stroke/TIA	20 (5.3)	32 (5.8)	1 (2.0)	3 (6.1)	0.743	0.595
MI, n (%)	36 (9.6)	22 (4.0)∗	6 (12.0)†	4 (8.2)	<0.01	0.036
Elective PCI, n (%)	30 (8.0)	12 (2.2)∗	2 (4.0)	1 (2.0)	<0.01	0.205
FU months	28 (15-45)	32 (17-49)	27 (10-47)	35 (18-42)	0.069	-

CABG, coronary artery bypass graft; FFR, fractional flow reserve; FU, follow-up; MACCE, major adverse cardiac and cerebrovascular events; MI, myocardial infarction; PCI, percutaneous coronary intervention; TAVI, transcatheter aortic valve implantation; TIA, transient ischemic attack.

∗ Different from the angio-guided PCI group, unadjusted *p* < 0.05.

† Different from the angio-guided no PCI group, unadjusted *p* < 0.05.

‡ Adjusted for age, gender, creatinine, previous MI, previous PCI, previous CABG, previous pacemaker.

### Angiography-Guided PCI vs. FFR-Guided PCI

**Figure 3:**
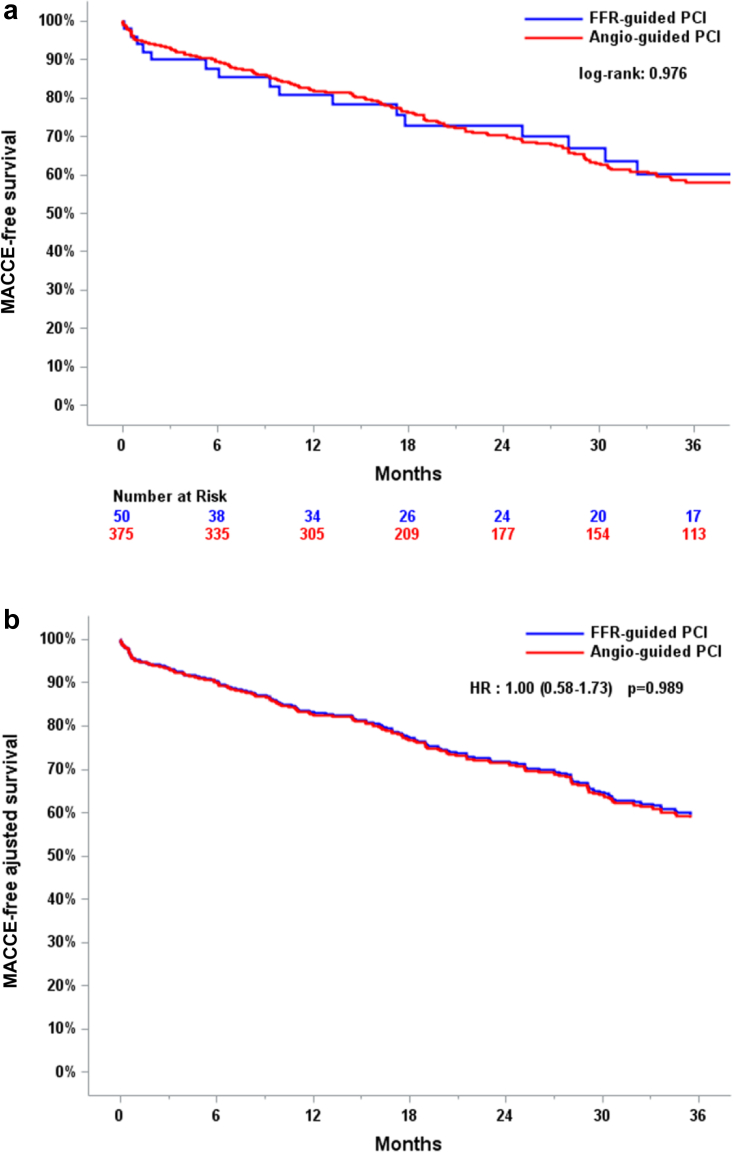
(a) MACCE-free survival and (b) MACCE-free adjusted survival for the primary outcome in the angiography-guided PCI group vs. FFR-guided PCI group. Abbreviations: FFR, fractional flow reserve; MACCE, major adverse cardiac and cerebrovascular events; PCI, percutaneous coronary intervention.

At long-term follow-up, the primary outcome occurred in 174 of 375 (46.4%) patients in the angiography-guided PCI group in opposition to 21 of 50 (42%) patients in the FFR-guided PCI group with an adjusted *p* value of 0.979 showing no statistically significant difference. MACCE-free survival Kaplan-Meier curve for this comparison is presented in Figure 3. For each individual component of MACCE, no significant differences were observed (Table 4, Table 5).

### Angiography-Guided PCI vs. FFR-Guided No-PCI (FFR-Guided Deferral)

**Figure 4:**
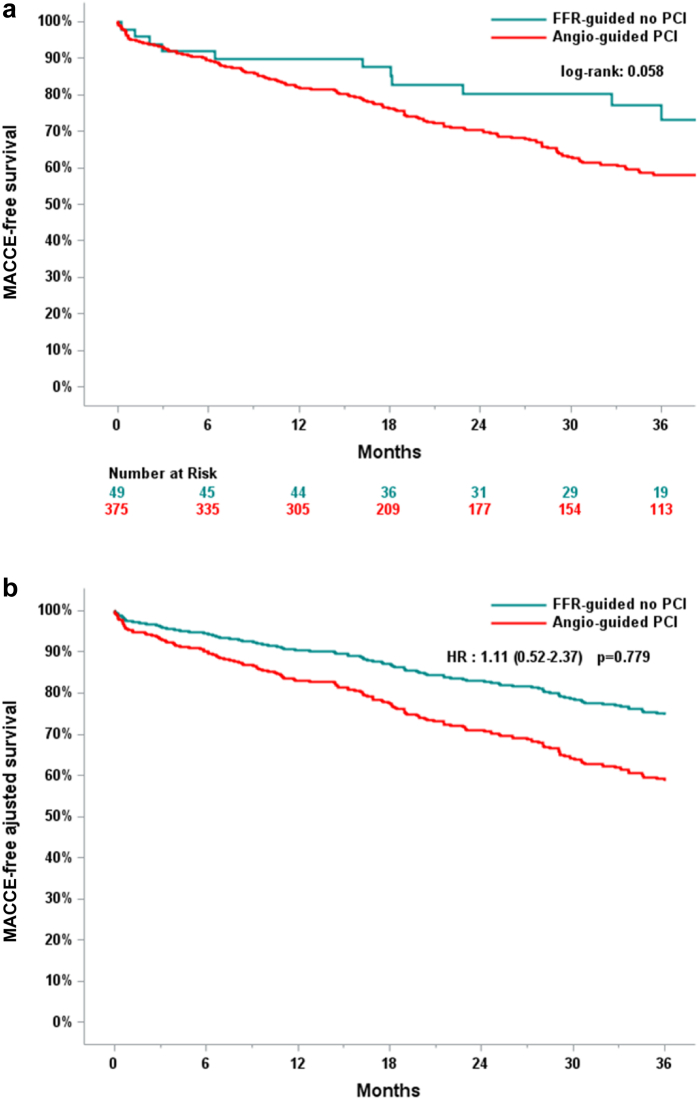
(a) MACCE-free survival and (b) MACCE-free adjusted survival for the primary outcome in the angiography-guided PCI group vs. FFR-guided no-PCI group. Abbreviations: FFR, fractional flow reserve; MACCE, major adverse cardiac and cerebrovascular events; PCI, percutaneous coronary intervention.

In this comparison, the primary composite outcome occurred in 174 of 375 (46.4%) patients in the angiography-guided PCI group in contrast to 16 of 49 (32.7%) patients in the FFR-guided no-PCI group (*p* = 0.999). The incidence of MACCE in the FFR-guided no-PCI group did not show statistical differences even though the curves tend to diverge (Figure 4). For this comparison, there were no statistical differences between the 2 groups in terms of the incidence of MI (2.1% vs. 3.5% *p* = 0.872), major bleeding (6.1% vs. 9.3% *p* = 0.511), major vascular complication (2% vs. 6.7% *p* = 0.281), or AKI (8.2% vs. 12.7% *p* = 0.061) (Table 4, Table 5).

### Angiography-Guided No-PCI vs. FFR-Guided No-PCI

The primary outcome occurred in 218 of 549 (39.7%) patients of the angiography-guided no-PCI group in comparison to 16 of 49 (32.7%) patients in the FFR-guided no-PCI group. No significant differences in terms of primary (*p* = 0.240) and secondary outcomes were demonstrated (Table 5).

### Adverse Events During Intracoronary Adenosine Infusion

Maximal hyperemia was achieved using intracoronary adenosine bolus in 91 of 99 patients in the FFR-guided group. The remaining 8 patients did not receive adenosine because their baseline pd/pa value was already ≤0.8. The mean total doses (including multiple measurements in the same vessel) were >200 μg for the left main and right coronary arteries. Only 1 patient had an adverse event during a 150-μg intracoronary adenosine bolus in his right coronary artery documented as rapidly self-resolving paroxysmal atrial fibrillation without hemodynamic instability. No other adverse events were documented otherwise.

## Discussion

The main findings of our study are that (1) in patients awaiting TAVI with CAD, an assessment with FFR resulted in no statistical differences in terms of long-term MACCE compared to an assessment with angiography alone (42.4% vs. 37.4% *p* = 0.333); and (2) when the decision was to proceed with PCI after FFR evaluation as opposed to following angiography alone (FFR-guided PCI vs. angiography-guided PCI), primary (42% vs. 46.4%, *p* = 0.979) and secondary outcomes showed no statistically significant differences. Studies have shown that the correlation between angiographic and hemodynamic significance of coronary lesions in patients with severe AS is modest and occurs mostly in cases of left anterior descending artery.^24^ LAD artery disease was present in 51.5% (Table 3) of patients in the FFR-guided groups and could explain our similar clinical results to an extent.

In patients who did not undergo PCI due to an angiography-guided strategy alone vs. those for whom this decision was confirmed with functional assessment (angiography-guided no-PCI vs. FFR-guided no-PCI), no statistically significant differences (39.7% vs. 32.7% *p* = 0.240) were observed in terms of primary outcomes. There were numerically more MI cases at long-term follow-up in the FFR-guided no-PCI group, but the statistical significance of this finding was not established (8.2% vs. 4%, *p* = 0.322). One plausible explanation could be the greater severity of lesions in the FFR-guided no-PCI group and the expected development of clinically significant CAD with time. Another tenable explanation is the possibility, although infrequent, of borderline coronary lesions to cross the FFR cutoff and become significant after valve replacement.^15^ Of note, only 2 patients who did not undergo PCI following FFR assessment developed an MI in the form of a non-ST-elevation myocardial infarction where the culprit lesions were ones initially deemed nonfunctionally significant. Although not statistically significant, this result could indicate the efficacy of FFR measurement at safely deferring PCI in a frail population awaiting a TAVI procedure.

Specifically, we believe the advantage of FFR measurements in patients awaiting TAVI lies in its capacity to defer intervention. The recently published ACTIVATION trial showed that (1) pre-TAVI PCI was associated with higher bleeding risks and that (2) the observed 1-year rates of mortality and rehospitalization were comparable between PCI and no-PCI prior to TAVI.^25^ PCI deferral of an intermediate coronary stenosis based on FFR in patients without AS is established as safe and favorable.^26^ Deferring coronary intervention following a functional assessment strategy showed promising results in another observational study of patients with severe AS.^17^ Avoiding the inherent periprocedural and postprocedural risks associated with PCI seems to justify the strategy of FFR-guided deferral. Unlike the observational study by Lunardi et al,^17^ we did not demonstrate a statistical difference between the occurrence of MACCE in the FFR-guided no PCI vs. angiography-guided groups at 33.7 months of follow-up (32.7% vs. 46.4%, *p* = 0.999). We believe that our study was probably underpowered to show a statistically significant difference in that instance. When looking at secondary in-hospital outcomes after TAVI, numerically lower rates of major bleeding, major vascular complications, and AKI were seen in the FFR-guided no-PCI group. A plausible explanation is that PCI deferral leads to less procedural adverse events (e.g., reduction of the bleeding risk associated with the avoidance of prolonged double antiplatelet therapy). It is also interesting to note similar procedural MI following TAVI in the angiography-guided and FFR-guided groups (25/924 vs. 3/99) (2.5% vs. 3%, *p* = 0.75), demonstrating the safety of deferring revascularization of functionally unsignificant lesions at the time of procedure.

Doses of IC adenosine greater than 150 μg were administered (Table 3), with literature showing a negligible FFR value variation when compared to intravenous access.^20^ Our study provides the largest disclosed data on the safety profile of intracoronary adenosine use in patients with severe AS. The occurrence of only 1 isolated rapidly resolving atrial fibrillation event in 1 patient provides reassuring safety data on intracoronary adenosine use in patients with severe AS.

### Limitations

First, our study included a relatively small number of patients. Also, owing to the nonrandomized, nonblinded observational nature of this study, no absolute conclusions can be derived from our results. The single center and longitudinal aspect of our study could also affect its external validity. Coronary artery lesion severity and localization were different in the angiography-guided and FFR-guided groups as the decision to proceed with functional assessment is usually made in a linear fashion with angiographic evaluation rather than a substitute or an alternate method. An underlying selection bias is therefore possible as the decision to proceed with FFR and/or PCI was left to the operator’s discretion. Nonetheless, our study represents real-life practice, and our results showed similar outcomes in patients where functional assessment allowed for deferral of PCI.

Another caveat of our study is the possible improvement of hyperemic whole-cycle coronary flow after TAVI.^16^ Patients in our study had an angiography as a part of the standard pre-TAVI workup. Thus, the decision to proceed with PCI following FFR was essentially made in patients with stable CAD, and no coronary physiological assessment was performed after TAVI. Nevertheless, the existing evidence shows that FFR variations after TAVI are minor and crossed the diagnostic cutoff of 0.80 in only 6% of patients after TAVI.^15^ The design of the study did not allow us to presume superiority of FFR compared to angiography as the decision to proceed with FFR was in sequence to angiography in most cases. We can only hypothesize that randomization would show similar results as those seen in landmark studies on FFR in patients without AS.^11^ Finally, optimal timing of percutaneous intervention is an area of ongoing research in FFR-guided revascularization and may limit extrapolation of our results.

## Conclusion

In conclusion, at 33.7-month follow-up, an FFR-guided strategy showed no statistically significant differences in terms of MACCE when compared to an angiography-guided strategy whether there was an ensuing PCI or not. The concept of FFR-guided deferral is interesting and could lead to fewer adverse events (MI, major vascular complication, AKI, major bleeding) by better selecting patients in whom PCI and its inherent risks can be avoided. Intracoronary adenosine use is safe and well tolerated for FFR assessment in a population with severe AS awaiting TAVI. Nevertheless, to help refine the role of FFR in the management of CAD in the setting of TAVI, further larger, randomized clinical trials will be necessary.

## Ethics Statement

This retrospective observational cohort study was approved by the ethics committee of the Quebec Heart and Lung Institute.

## Funding

This research received no specific grant from any funding agency in the public, commercial, or not-for-profit sectors.

## Disclosure Statement

Dr Josep Rodés-Cabau has received research grant support from 10.13039/100006520Edwards Lifesciences, 10.13039/100004374Medtronic, and 10.13039/100006279St. Jude Medical. The other authors had no conflicts to declare.
